# Play as practice? Comparative analysis of preparation period and match adjustments in a basketball team’s performance

**DOI:** 10.1371/journal.pone.0312678

**Published:** 2024-10-29

**Authors:** Alan Monteiro, Matthew Heiner, Gilbert Fellingham, Leonardo Lamas

**Affiliations:** 1 LabEsporte, Faculty of Physical Education, University of Brasilia, Campus Darcy Ribeiro, Brasilia, BR; 2 Department of Statistics, Brigham Young University, Provo, Utah, United States of America; Instituto Politecnico de Santarem Escola Superior de Desporto de Rio Maior, PORTUGAL

## Abstract

In basketball, successful performance relies on the optimal use of top-down strategic guidance by coaches and bottom-up adjustments by players, requiring a preparation plan consistent with match demands. The aim of this study was to analyze the strategic-tactical tendencies between a basketball team’s preparation and match performance phases for a U16 men’s national team during a continental tournament. The team was composed by 12 players (aged 16 ± 0.4) with at least three years of participation in basketball competitions. Data from team strategy (the playbook), team practices, and match performance were integrated through a common set of variables in a decision support framework, the Team Learning Cycle (TLC). The influence of situational variables with respect to preparation and match performance consistency and efficiency was also investigated. The preparation phase significantly emphasized small-sided games focused on group-tactics-based attacks, combining offense and defense, usually in the set offense. During the matches, the set offense was also significantly prioritized. The frequency of group-tactics-based attacks increased relative to team plays from the group phase to the elimination phase (*p* < 0.05). Efficiency generally improved during close matches, suggesting successful team strategy learning and tactical autonomy from preparation to matches. Using consistency and efficiency together provided for an effective evaluation of a team’s preparation-performance relationship. This evidence underscores the importance of sensitive monitoring methods for establishing accurate associations between preparation and performance. Coaches can use this systematic procedure to critically examine their use of preparation time relative to match performance. Additionally, basketball managers may find that TLC-related evidence supports evaluating coaches’ multi-dimensional skills from a broader perspective than simply winning rates, offering a more objective and comprehensive assessment of coaching effectiveness.

## 1 Introduction

In basketball, team strategy is essential for fostering team play and enhancing performance. However, the effectiveness of executing these strategies is often hindered by the opponent’s ability to anticipate actions, aided by various scouting techniques. Consequently, player creativity becomes vital in introducing unpredictability into the game, thereby gaining a performance edge [1]. Success against the opposition hinges on the optimal integration of top-down strategic guidance with bottom-up tactical adjustments made by the players [2]. The strategic-tactical connection leverages the potential interactions among the five team players over the 40 minutes of effective play, enhanced by the flexibility of an unrestricted number of player substitutions.

The ability to choose the optimal strategic-tactical solutions is not innate to players, making it the coach’s responsibility to design preparation plans that enhance both individual and collective performance [3, 4]. This is a non-trivial task that can benefit from decision support frameworks [5]—computer-based information systems that provide objective evidence of environments where the available data are vast and exceed human processing capabilities [5, 6]. This is particularly relevant in basketball, where the literature acknowledges the need for practical implementations to monitor the relationship between preparation and performance [4, 7].

Methodological [8] and technological [9] advancements have significantly improved the ability to compute and retrieve data from training practices, supporting a team’s performance [3]. Recently, a more comprehensive approach has been proposed through an integrative framework encompassing the main steps of the team preparation-performance process [10]. This framework, known as the Team Learning Cycle (TLC), includes team planning (i.e., strategy design), the teaching-learning process (i.e., team practices), and match performance, in cycles that restart after each match and continue throughout a basketball tournament or season [10].

The TLC integrates strategic, tactical, and technical components across all preparation and performance stages. Its use provides evidence of how top-down inputs (implementation of team strategy through practice) and bottom-up tactical solutions from matches complement each other in team performance [2]. Initial results from Rangel et al. [10] indicate that the emphasis on certain strategic-tactical contents during practice did not always correspond to those observed during matches. However, some practice emphases did achieve high efficiencies in match performance. Inconsistencies between preparation and performance indicate that non-obvious associations are required from the coach, reinforcing the need for appropriate monitoring methods.

Implementation of the TLC [10] successfully addressed the methodological gap by providing a framework for comparing preparation and match performance. Nonetheless, empirical evidence has been limited due to the assessment of only a single match. Generally, tactics observed in a match are influenced by the adversary and situational variables, such as the tournament phase [11, 12]. To better understand strategic and tactical adjustments, investigations should consider multiple matches. The present study adds to the current literature by applying a TLC-based assessment considering multiple matches. This should provide a more comprehensive assessment of the team’s consistency and efficiency in adhering to the plan while making necessary adjustments based on match circumstances.

The aim of the present study was to analyze the preparation-performance process of a basketball team by comparing the strategic-tactical tendencies between the preparatory phase and actual performance across multiple matches. Specifically, the efficiency of top-down strategic inputs and bottom-up player tactics within matches were examined. The hypothesis was that tactical adjustments would increase relative to strategy-based play structures (i.e., team plays) as the tournament progressed to the decisive phase. This could indicate performance optimization attempts as players improve their cooperation and decision-making abilities [13, 14]. Consistency between preparation-performance and efficiency could be maintained if the preparation period fostered strategic and tactical learning [15].

## 2 Materials and methods

### 2.1 Study design

This was a quantitative, observational, longitudinal study. Consistency and efficiency between preparation and performance were analyzed with a U16 national men’s basketball team during the matches of a short-term, high-level tournament. Data from team strategy (the playbook), team practices, and match performance were integrated. A previously defined framework for integrative evaluation of team preparation and performance in basketball—i.e., the TLC—was followed [10].

Analysis was performed within each main step of the TLC. The team strategy was evaluated on its sequences of space creation dynamics—SCDs [16, 17]. The training practices were assessed through pedagogical variables to discriminate the methodological approach used by the coach to improve individual, group, and collective performance [8]. Match tactics (tendencies and efficiency) were analyzed during six tournament matches.

Data were used to compare tendencies found in the team preparation and in the match performance, including: i) offensive sub-phases (i.e., transition game, set offense); ii) offensive structures (i.e., team plays, SCDs). Match events were discriminated according to the following situational variables: i) tournament phase (group phase, elimination phase); ii) match period (first and second half); iii) point spread (≤ 10 points, > 10 points).

### 2.2 Participants

Participants comprised the Brazilian U16 national men’s basketball team during the 2019 America Cup. There were 12 players (aged 16 ± 0.4) with at least three years of participation in basketball competitions. The head coach had a bachelor’s degree in sports sciences, a master’s degree in sports sciences, and ten years of experience as a basketball coach. The team performed all training practices before the tournament started. In the tournament, the team competed in six matches. In the group phase, the matches and outcomes were: Brazil 72 x 58 Puerto Rico; Canada 90 x 67 Brazil; Brazil 55 x 68 Uruguay. In the elimination phase: Dominican Republic 73 x 71 Brazil; 5th—8th place: Brazil 64 x 62 Mexico; 5th—6th place: Puerto Rico 61 x 78 Brazil.

### 2.3 Procedures

The team’s head coach provided all the required resources to support the empirical analysis of the TLC steps. The team strategy was assessed from the season’s playbook with sequential diagrams of each team play, including the players involved and the location on the court where the play occurred. The team strategy was broken down based on the plays and their respective SCDs (i.e., 1x1, cut, handoff, pick, post-up, screen, spot-up), following previously defined criteria [16].

Practice data were gathered from the period of the team preparation. Twenty training sessions were performed during two weeks (77.5 minutes, on average, per practice). The coach’s teaching-learning methods were assessed through the time (in minutes) allocated to the following pedagogical variables [8]: i) match phases—offense, defense, mixed; ii) content types—strategic (video-based or on-court contents, focused on the understanding of the team strategy), tactical (individual, group, collective, with opposition and performed within the match context), technical (individual, focused on technical skills improvement); iii) training means—drill (analytic task, without opposition, with emphasis on game-related technical skills, e.g., shooting), “walk-through” (on-court, collective, without opposition, focused on adjustments of spacing and timing of team plays), game (situational tasks, with emphasis on individual or group tactics), competition (situational tasks, with emphasis on collective tactics); iv) game situations (the numeric configurations of the opposition)—1x0–1 (one versus zero defenders or one defender), 2–3x0, 2–3xN (where N represents any number of defenders), 4–5x0, 4–5xN. Minutes of practice were computed for the following breakdown of the team offense: i) offensive sub-phases—transition game, set offense; ii) offensive structures—team plays, SCDs. These data were used for comparisons among matches.

Match performance was analyzed in all six matches of the tournament. Matches occurred immediately after the end of the training period. In each match, the following variables were computed for every ball possession: i) the offensive sub-phases—transition game, set offense; ii) the offensive structure used in the possession to achieve the outcome—team plays, SCD; iii) the efficiency, in points per possession, given the outcome observed (two points converted/missed, three points converted/missed, shooting foul, defensive foul, offensive foul, turnover).

A single researcher (AM) collected the data from all TLC steps. The researcher’s reliability in notating the offensive sub-phases, offensive structures, and outcomes in ball possessions was previously assessed. Inter-observer reliability assessment was also performed. To evaluate the intra- and inter-rater observers’ reliability, a set of 70 ball possessions from a top professional men’s basketball match was assessed and the same variables were annotated. The procedure was performed on two different occasions, two weeks apart, following recommendations found in related literature [18, 19]. Reliability scores were evaluated according to the levels of agreement for the Cohen’s Kappa values [20], with the following scale: < 0 no agreement, 0.01—0.20 slight agreement, 0.21—0.40 fair agreement, 0.41—0.60 moderate agreement, 0.61—0.80 substantial agreement and 0.81—0.99 almost perfect agreement [20]. Kappa values ranged from 0.91 to 1.0 for all variables, indicating almost perfect agreement in intra-rater assessments (offensive sub-phases: 1.0; offensive structures: 0.94, and outcomes: 1) and inter-rater assessments (offensive sub-phases: 1.0; offensive structures: 0.91; and outcomes: 1.0). Methods applied in this study were in accordance with procedures approved by the Institutional Review Board of Brigham Young University.

### 2.4 Data analysis

Descriptive and inferential analyses were used to evaluate the TLC steps and the associations between them. Descriptive statistics were used to characterize the structure of the team’s offensive strategy as outlined in the playbook considering the number of plays, the SCDs in each play [17], and the location on the court where the SCDs were planned.

Bayesian methods were used to estimate each training variable’s mean number of minutes used during practice. Since the distribution of time variables was right-skewed, a gamma distribution was used for the likelihood of these variables. Fairly non-informative priors were used for the parameters of the likelihood distributions. Posterior distributions of differences between means of the training variables were approximated using differences of the Markov chain Monte Carlo (MCMC) draws for the parameters. When evaluating inferences for every comparison, the term “significantly different” was used when the posterior probability (p.prob) of the difference exceeding 0 was > 0.90. The computer program JAGS [21] was used to simulate chains approximating the posterior distributions of interest.

For the match, a logistic regression was used to model the proportions of the offensive structures (team plays and SCDs) used by the team in the tournament. The factors used to account for differences in this response were the following situational variables: i) tournament phase (group and elimination); ii) score spread ≤10, >10; iii) match period (first, second half). The optimal model was selected using the Akaike Information Criterion (AIC) [22]. A Chi-square test was conducted to assess differences in the team plays’ usage between the group phase and the elimination phase of the tournament, at an alpha (significance) level of 0.05. All statistical procedures were performed using R software (Core Team, Vienna, Austria. Version 4.3.0) [23].

## 3 Results

**Fig 1:**
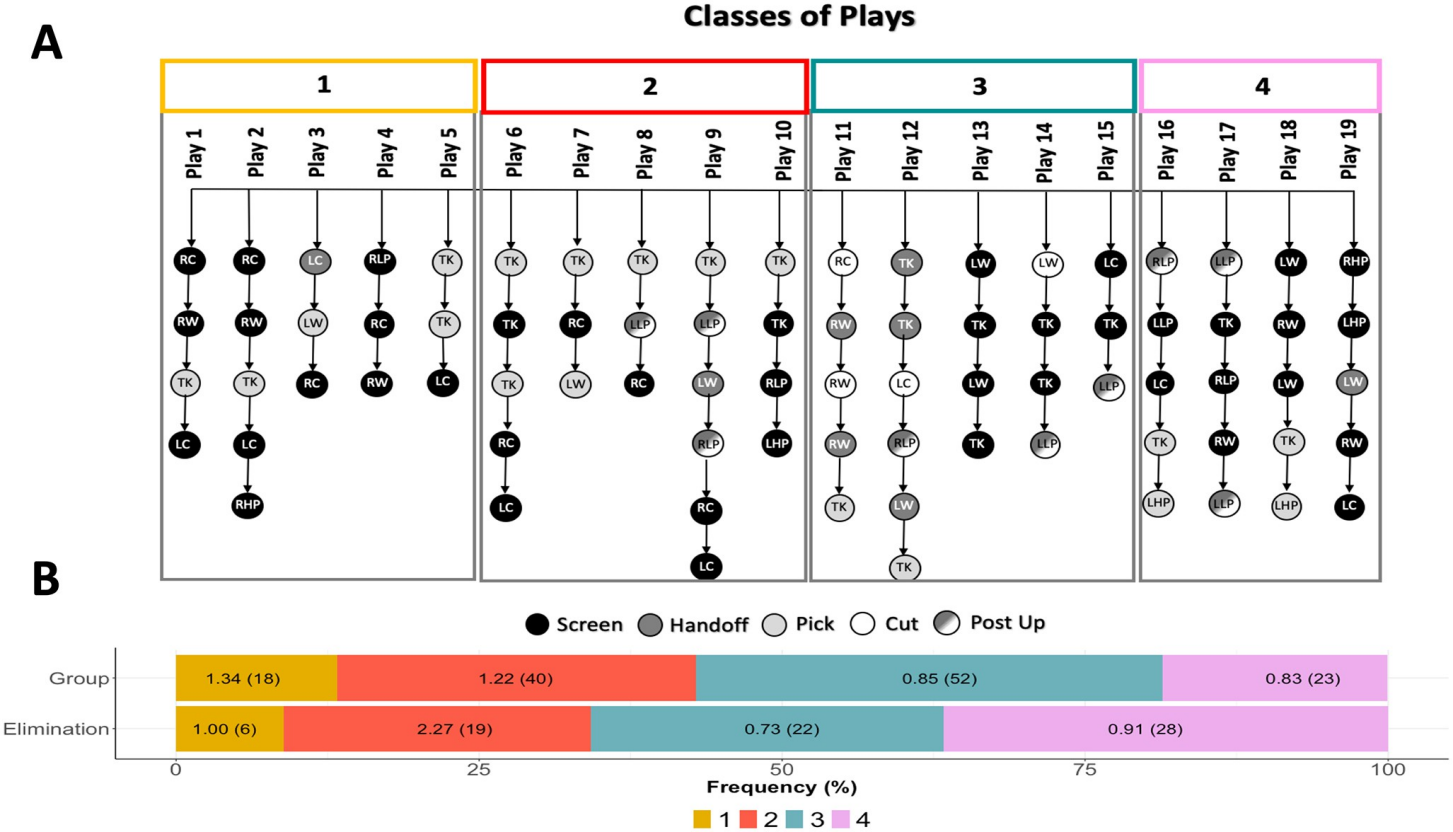
The team strategy: Structure and match usage. A: Team strategy’s classes of plays, where a class of play, from 1 to 4, is defined by a set of similar plays with variants among them. Each branch of nodes is an individual play. In a play, each node is defined by its main SCD. The SCD is assigned by the node’s color, in grey scale. The SCD location on court is indicated inside the node, with TK representing top key; RW right-wing; LW left-wing; RC right corner; LC left-corner; RHP right-high-post; LHP left-high-post; RLP right-low-post; LLP left-low-post. B: Relative frequency of each play class in the group and elimination phases of the tournament. Efficiency and absolute frequencies inside bars.

The team strategy was designed with 19 plays, organized into four classes (see Fig 1—Part A; numbered 1—yellow, 2—red, 3—green, 4—purple). In the whole team strategy, there was a predominance of screens (43; 53%), followed by picks (17; 21%), post-ups (9; 11%), hand-offs (8; 10%) and cuts (4; 5%) within plays. The colors assigned to each play class correspond to those of the stacked bars in Fig 1—Part B. In Part B, bars display the proportions of classes of plays performed in the group and elimination phases, respectively. Values inside bars report efficiency, in points per possession, and absolute frequencies.

**Fig 2:**
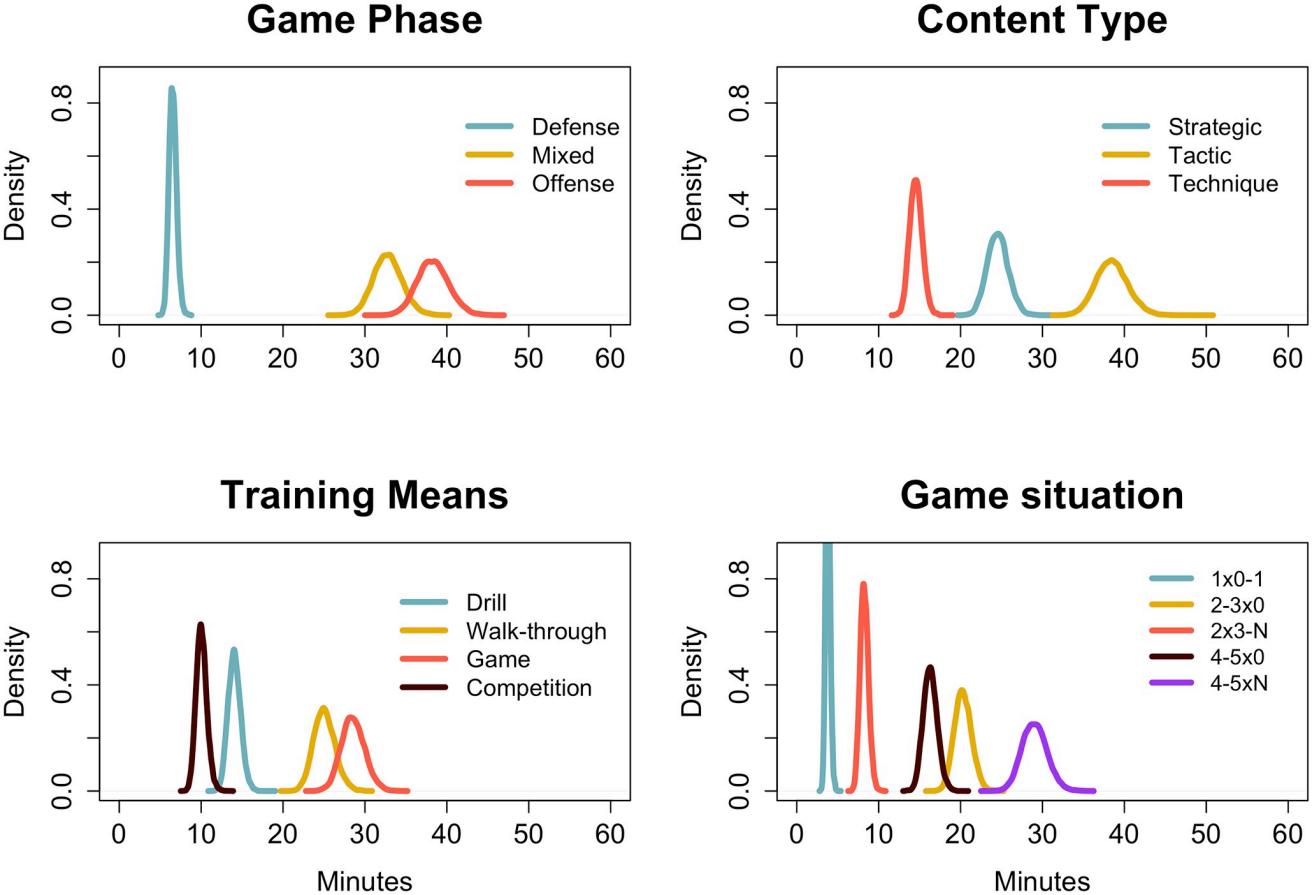
Posterior distributions of mean practice time of four pedagogical variables. Game phase, content type, training means, and game situation during the preparation period.

The contents emphasized by the coach during the team practices were computed according to four pedagogical variables (see Fig 2). For the variable game phase, offense and mixed (offense and defense combined) were practiced for significantly more time than defense (p.prob > 0.90). The tactics content type was emphasized significantly more frequently than strategy and technique. For the training means, game (defined by the several possibilities of small-sided games (SSGs)) and the walk-through of plays were practiced for significantly more time than drills and competition (i.e., scrimmages). Finally, for the game situations, 4–5 players versus any number of players was the most frequent situation. Together with 2–3 versus none and 4–5 versus none, all three were practiced significantly more frequently than 2x3 versus any number of players and 1x1 or none.

**Fig 3:**
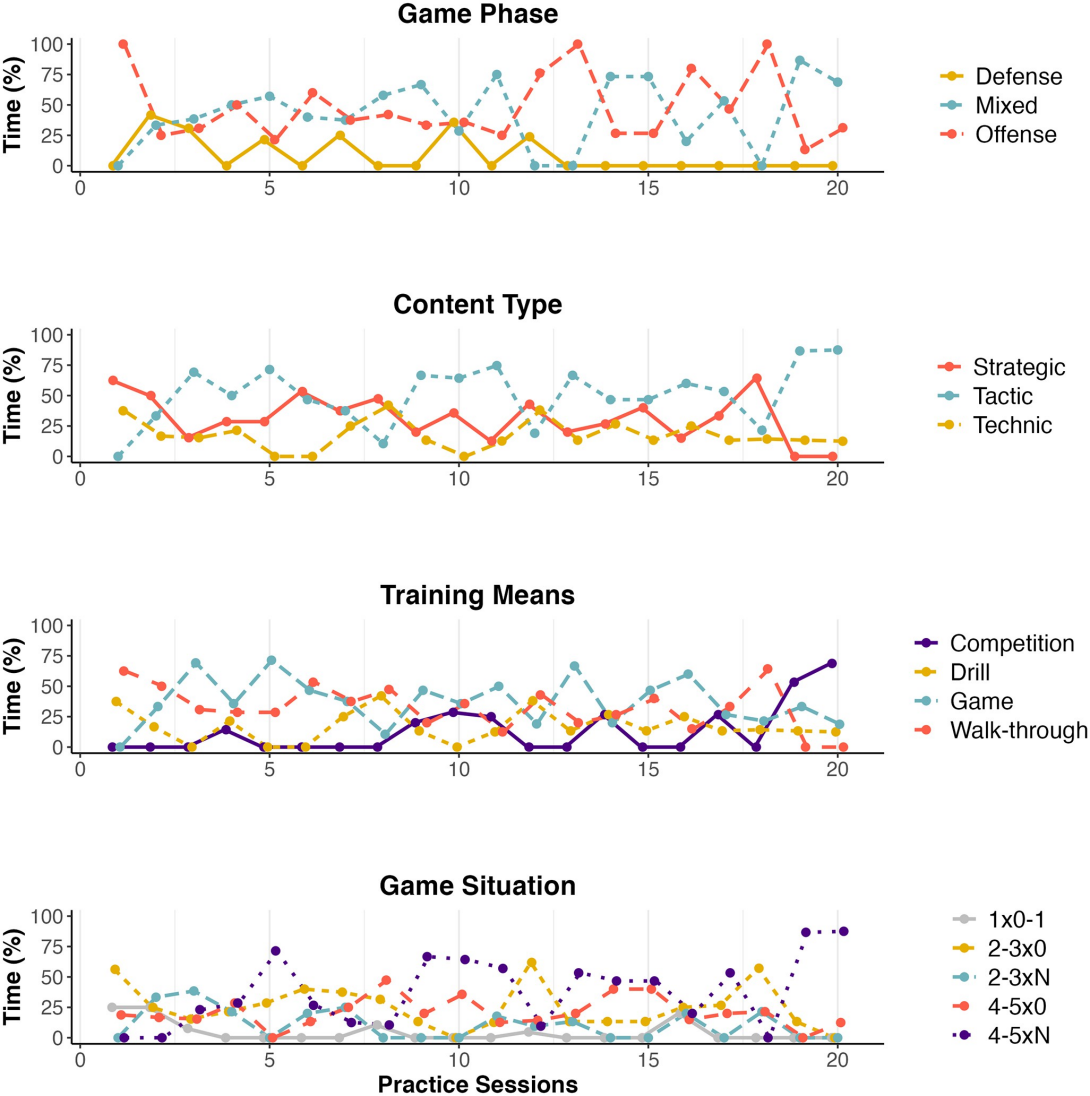
Daily variability of pedagogical variables within the preparation period.

The match phases offense and mixed were alternately prioritized daily. Regarding content type, tactics were emphasized throughout the training period while strategy and technique were moderately practiced. When practice focused on strategy, it was synchronized with the game phase offense. Still, content types strategic, tactic, and technique corresponded mainly to, respectively, the training means walk-through, game/competition, and drill. Finally, game situations were mostly performed with SSGs with 2–3 attackers versus zero defenders or games with a structure more similar to the formal game (4x5 versus N), see Fig 3.

**Fig 4:**
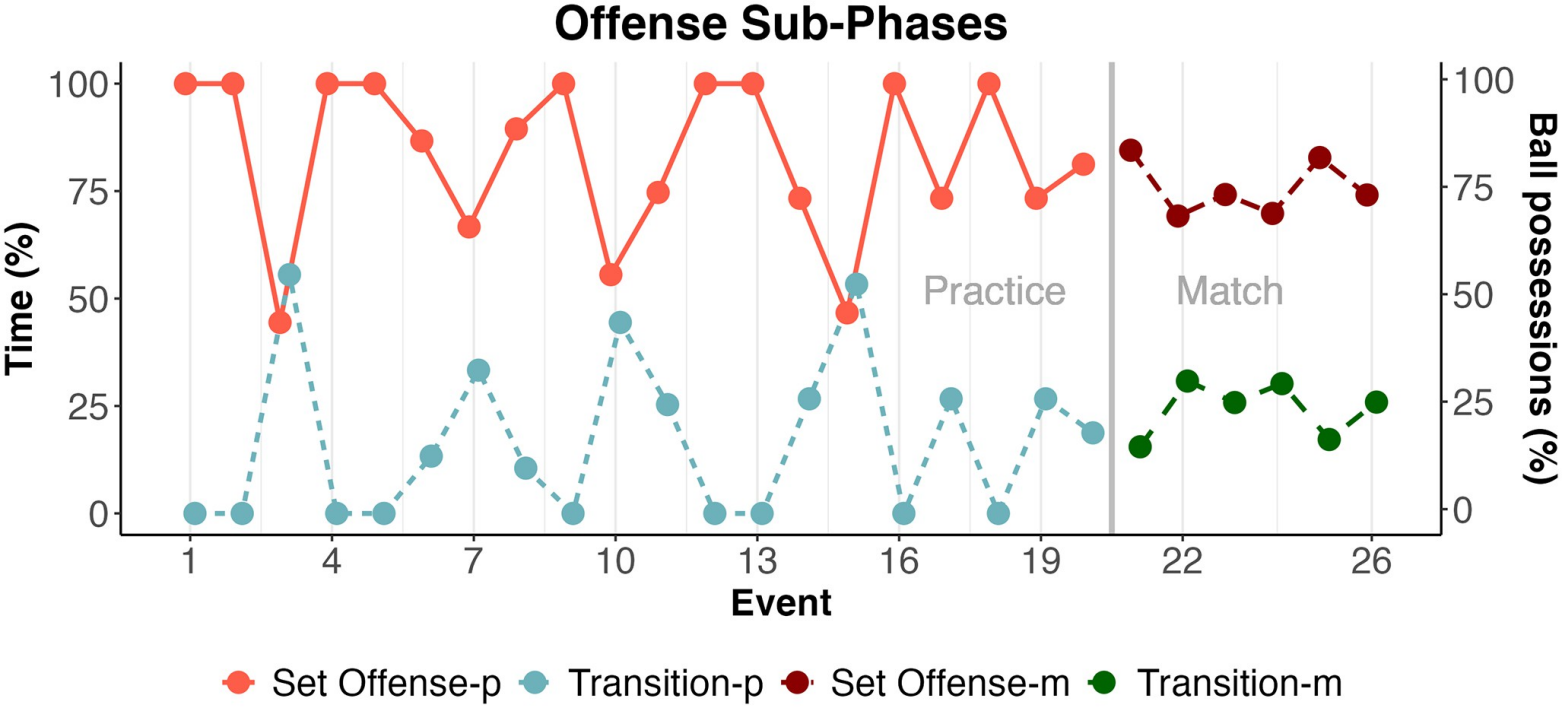
Comparison of offensive sub-phases: Proportion of the offensive sub-phases (set offense, transition game) computed, respectively, in minutes during the training period (-p) and the number of ball possessions during the six matches (-m).

The comparison between practices and matches first considered the emphasis given to the offensive sub-phases. The set offense was emphasized significantly more frequently than transition game both in the practices and in the matches (see Fig 4).

**Fig 5:**
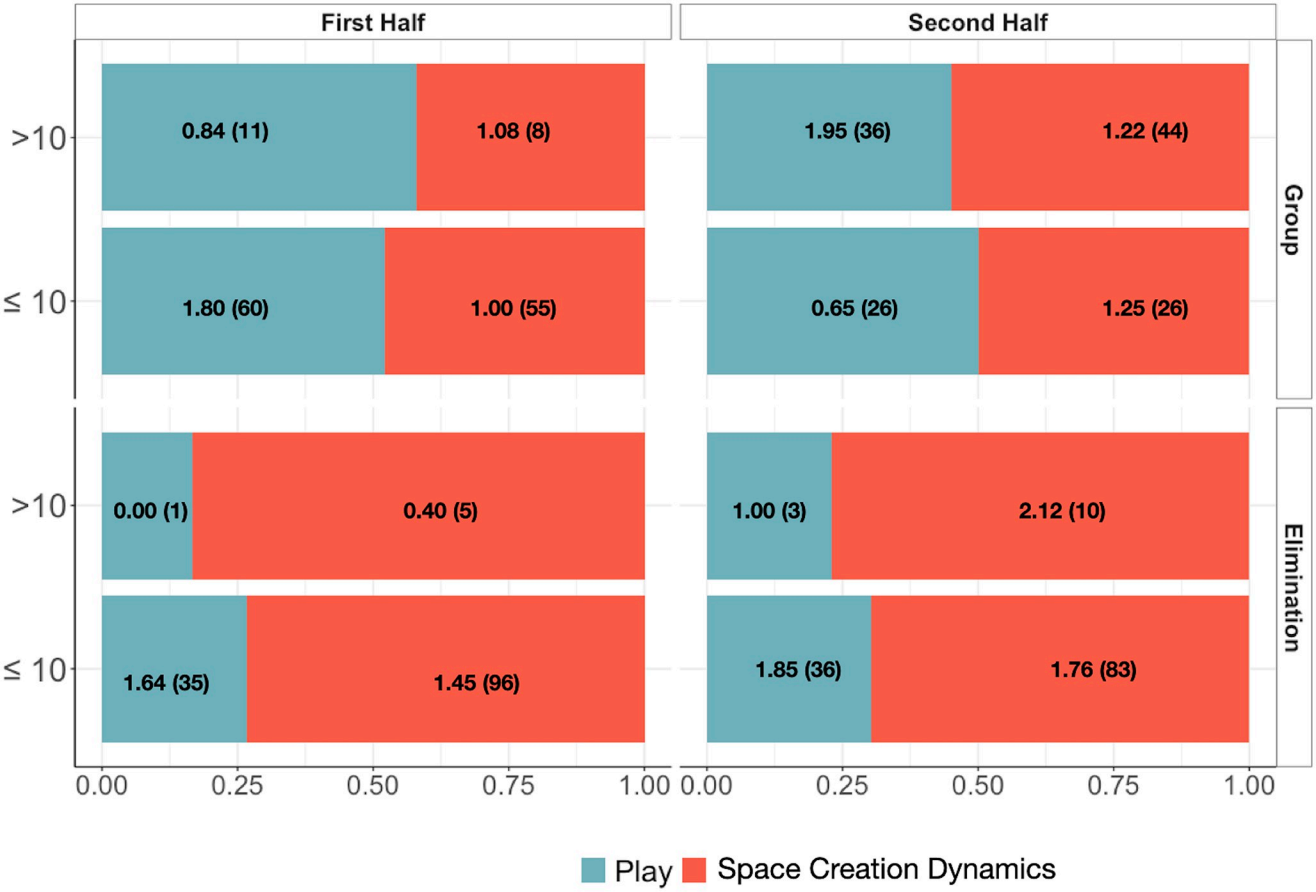
Proportions of team plays and SCDs in the match periods (first half, second half); point spreads (≤10, >10); tournament phases (group, elimination): Efficiency, in points per possession, appear inside bars along with absolute frequencies.

The practice and match comparison also considered the association between the offensive structures (team plays or SCDs) and each of three situational variables, i.e., tournament phase (group phase, elimination phase), match period (first or second half), and point spread (≤10 points or >10 points), in each match. Logistic regression results indicated that only the variable tournament phase was significantly associated with proportions of team plays and SCDs (z-value = -5.19, *p* < 0.001). The model used to obtain this result included only the main effects since the interaction terms were not significant. See Fig 5.

Variability in team plays was analyzed by their frequencies according to the tournament phases (Fig 1—Part B). Chi-square test results indicated significant differences between the group and the elimination phases of the tournament (*χ*^2^ = 8.9768, *df* = 3, *p* = 0.0296). Standardized residuals indicated a significant increase in the use of play class number 4 from group to elimination phase (2.96).

## 4 Discussion

This study aimed to compare the strategic-tactical tendencies between a basketball team’s preparation and match performance phases. The main findings related to each step of the TLC revealed that the four play classes of the team strategy had similar game actions but with particular goals according to their distinct criteria of concatenation. Additionally, the preparation phase emphasized SSGs, focused on SCDs, frequently combining offense and defense, usually in the set offense. During the matches, the set offense was equally prioritized. SCD-based attacks increased from the group phase to the elimination phase relative to team plays, and efficiency generally improved during close matches, suggesting both team strategy learning and tactical autonomy acquisition. Consistency and efficiency combined for an effective evaluation of a team’s preparation-performance throughout the TLC.

The team strategy was broken down following a set of previously defined criteria [16]. Quantitative analysis of basketball team strategies adds evidence to its features, often explored based on common-sense perspectives [24–26]. Particularly, investigations on team strategy supported by the TLC may yield indicators of a coach’s strategic performance, enabling comparisons of their strategic conceptualization, communication during practices, and ultimately its execution during matches.

The team strategy currently analyzed was designed with four classes of plays, following a previous analytic approach for assessing basketball strategies [10, 16]. Classes of plays are differentiated mainly by the players’ starting positions and sequences of game actions (SCDs). According to Fig 1, in a play class, both the SCDs (grey scale of the nodes) and SCD location on the court (label inside each node) vary among plays, adding unpredictability and configuring variations for the given play. Distinct classes of plays present particular offensive principles and were designated differently by the coach (i.e., classes 1 to 4). Team play number two, for instance, started all its variations at the top of the key (Fig 1, inner label—TK) with a pick (Fig 1, light grey node), which is characteristic of a well-known offensive configuration in basketball (i.e., the horns series or pick and roll). Given the short preparation time, this may have been an option to aid the players in learning the team strategy. The screen was the most frequent SCD (53%) in the team strategy, corroborating the result of Rangel et al. [10]. This suggests a dynamic game style, with collective space creation, instead of a style centered on one or a few players. An analysis based on game actions complements previous contributions whose assessments used box-score indicators (e.g., rebounds, assists, etc [26] or consisted of more theoretical approaches [27].

There were 20 sessions for preparing the U16 team for the tournament, resulting in a highly time-constrained context for implementing the game model [28]. Four pedagogical variables [8, 29] were chosen to assess the coach’s teaching-learning alternatives and used to compare preparation and match performance. Game phases offense and combined were significantly more emphasized than the defense. Practice was mainly focused on tactics, with SSGs used for this purpose. SSGs alternated between contexts with no opposition (2–3x0; 4–5x0) and “live” contexts (4–5 versus different numbers of players, 4–5 x N) to learn the team strategy. This approach facilitated players’ tactical learning as well. Still, results reinforce the contribution of the pedagogical variables to the evolving framework of tactical monitoring [30].

Daily practice data gave evidence of the enhancement of specificity during the period. There was a gradual increase in time spent on the offense-defense combination, through scrimmages and SSGs, with more than three opposing players during the practice period. There was also a greater focus on offense during the final practices. Tactics were prioritized during the whole period. The walk-through of team plays was also frequent since memorization of team strategy may be challenging in short-term preparation. Data highlight the effort to optimize time considering the compromise between stimulating problem-solving through tactics and ensuring the team strategy learning by the players. Generally, results point to similar tendencies of previous evidence based on the TLC, indicating the coach’s awareness in terms of emphasizing tactical learning [10]. The coach likely combined the presentation of the strategic-tactical contents in a less complex environment without opposition (2–3x0), progressing to contextualization (4–5x0) and application in “live” conditions (4–5xN). Thus, daily variability of practice demonstrated the segmentation of the team strategy into live conditions, possibly contributing to the players’ autonomous adjustments during the matches. These pieces of evidence add to other perspectives that combine for monitoring the teaching-learning and resulting performance throughout the preparation-performance process [31].

Previous concerns related to preparation-performance monitoring [3, 4] were approached by comparing team strategy, practice, and match performance. The implemented approach connects to the current discussion about descriptive-predictive monitoring tendencies in the field [30, 32], complementary to contributions focused on the conditioning domain [33, 34]. Our study found that proportions between offensive sub-phases were consistent between practices and matches (see Fig 4). In both practice and match, the set offense was utilized significantly more frequently than the transition game. The set offense was performed in 70–80% of ball possessions in every match, representing additional evidence of consistency, despite the opponent’s aims. The comparative analysis was possible because practice and match were assessed using similar structures [10].

Within the set offenses, there was a significant change in the offensive structures (i.e., team plays or SCD-based offenses) between competition phases (i.e., group and elimination). During the elimination phase, the frequency of SCD-based offenses increased (see Fig 5). The team increased unpredictability through bottom-up tactical adjustments, possibly in response to scouting during the group phase. These results add to previous evidence of modifications in the patterns of interaction among team players throughout the competitive period [31].

Indeed, the preparation-performance comparison showed that practices promoted strategic understanding and tactical enhancements. This balance was assessed using the efficiency of both offensive structures. In the elimination phase, both the team plays and SCD-based offense generally increased efficiency. Efficiency results indicate an appropriate alternation between top-down and bottom-up combinations of offensive structures during matches [35, 36].

The relative frequencies of the classes of team plays varied from group to elimination phase (see Fig 1, Part B). There was a significant increase in the use of plays from group 4. The relative frequency of group 2 plays was essentially unchanged. These plays included the pick and roll at the top of the key as a main action, a frequent offensive maneuver in basketball [37, 38]. Group 4, a set of special plays designed for close matches and end-of-game circumstances, was used more frequently during the elimination phase. Therefore, the tendencies among team plays were consistent with the tournament phase.

This study had certain limitations. Although the design incorporated multiple matches, it did not account for the preparation-performance dynamics typical of a basketball season, where practices and matches alternate. Consequently, the evidence presented does not reflect the impact of matches on subsequent training sessions or how those sessions influence future games. Furthermore, a typical season spans several months, producing a larger volume of data from both practices and matches. The study also had a limited sample size, consisting of twelve players from a single team, with an average age of 16 and at least three years of competitive basketball experience. Future research could address these limitations by including professional teams, where more complex strategic-tactical structures are implemented. Nevertheless, the current setup offers valuable insights into a relevant real-world scenario, particularly in the context of short-term national team tournaments.

## 5 Conclusion

The comparative analysis of preparation and performance showed that, despite the time constraints of the short practice period, achieving an effective balance between strategic and tactical content resulted in consistent and efficient performance throughout the tournament matches of an under-16 men’s basketball team. This evidence underscores the importance of sensitive monitoring methods [28], such as the TLC, for establishing accurate measurement of the relationship between preparation and performance. Coaches can use this systematic procedure to critically examine their use of preparation time relative to match performance. Additionally, basketball managers may find that TLC-related evidence supports evaluating coaches’ multi-dimensional skills from a broader perspective than simply winning rates, offering a more comprehensive assessment of coaching effectiveness.

The practical applications of the study relate to the integration of each TLC step. The proposed assessment of the team strategy supports the evaluation of the strategic diversity, considering the type of game actions specified and their concatenations in each team play. Additionally, Fig 1 summarizes the whole playbook with a single chart and includes a comparison of strategy usage during matches (Fig 1, Parts A and B). Team practice assessment was supported by a well-established set of pedagogical variables, allowing consistent monitoring of temporal training trends for the coach (see Fig 3). Finally, the TLC framework supported the comparison of the preparation process with the match tendencies, in terms of emphasis on game phases and offensive structures performed. This should be helpful to a coach’s evaluation of their decision process.

## Supporting information

S1 File (ZIP)
